# Supplementary material for: Deletion of Gadd45a Expression in Mice Leads to Cognitive and Synaptic Impairment Associated with Alzheimer’s Disease Hallmarks
Source: Int J Mol Sci. 2024 Feb 23;25(5):2595. doi: 10.3390/ijms25052595 (PMC10931605; doi:10.3390/ijms25052595)
Supplement: Supplementary file 1 [file ijms-25-02595-s001.zip › Table S1_primersGADD45.pdf]

Table S1. Syber Green Primers used in qPCR studies.

TARGET	FORWARD PRIMER (5'-3')	REVERSE PRIMER (5'-3')
Trem 2	CCTGAAGAAGCGGAATGGG	CTTGATTCCTGGAGGTGCT
Arg1	GTGGAGAAAGACATTCCAAGGC	CAGTTCAGGGATCTTGTACCCA
iNOS	GGCAGCCTGAGAGACCTTTG	GGAAGCGTTTCGGGATCTGAA
Nt3	CAGGGTGAAGGGGAAAACTC	AGTTCGGTCATTCAGTCTCGC
Bdnf	TGCGAGTATTACCTCCGCCAT	TCACGTGCTCAAAAGTGTCAG
Ngf	GGAGCGCATCGAGTGACTT	CCTCACTGCGGCCAGTATAG
TrkA	CTCCTTCTCGCCAGTGGAC	TGCCCTCAGTAGGGGAAAGA
TrkB	CGTCACTTCGCCAGCAGTAG	CTATACGCCAGGCACCACTC
β-Actin	CAACGAGCGGTTCCGAT	GCCACAGGTTCCATACCCA
