# Supplementary material for: Deletion of Gadd45a Expression in Mice Leads to Cognitive and Synaptic Impairment Associated with Alzheimer’s Disease Hallmarks
Source: Int J Mol Sci. 2024 Feb 23;25(5):2595. doi: 10.3390/ijms25052595 (PMC10931605; doi:10.3390/ijms25052595)
Supplement: Supplementary file 1 [file ijms-25-02595-s001.zip › Table S2_GADD45.pdf]

Table S2. Antibodies used in Western blot studies.

Antibody	Host	Source/Catalog	WB dilution
**GAPDH**	Mouse	Millipore/MAB374	1:2000
**Tubulin**	Rabbit	Abcam/ab15246	1:1000
**p-GSK3β ser9**	Rabbit	Cell Signalling/9336	1:1000
**GSK3β**	Rabbit	Cell Signalling/9315	1:1000
**p44/42MAPK**	Rabbit	Cell Signalling/9102	1:1000
**p-p44/42MAPK**	Rabbit	Cell Signalling/9101	1:1000
**p-Tau S396**	Rabbit	Invitrogen/44-752G	1:1000
**p-Tau S404**	Rabbit	Invitrogen/44-758G	1:1000
**Total Tau**	Rabbit	Gene Tex/112981	1:1000
**p-NF-KB**	Mouse	Santa Cruz/136548	1:1000
**Total NF-KB**	Rabbit	Cell Signalling/D14E12	1:1000
**PSD95**	Rabbit	Gene Tex/133091	1:1000
**p-ULK1 S757**	Rabbit	Cell Signalling/6888	1:1000
**Total ULK1**	Mouse	Santa Cruz/390904	1:1000
**Beclin-1**	Rabbit	Cell Signalling/3738	1:1000
**Goat-anti-mouse HRP conjugated**		BioRad/170-5047	1:2000
**Goat-anti-rabbit HRP conjugated**		BioRad/170-6515	1:2000
