# Supplementary material for: Deletion of Gadd45a Expression in Mice Leads to Cognitive and Synaptic Impairment Associated with Alzheimer’s Disease Hallmarks
Source: Int J Mol Sci. 2024 Feb 23;25(5):2595. doi: 10.3390/ijms25052595 (PMC10931605; doi:10.3390/ijms25052595)
Supplement: Supplementary file 1 [file ijms-25-02595-s001.zip › Table S3 GADD45.pdf]

Table S3. Parameters measured in the Open Field Test (OFT) in GADD45A$^{-/-}$ mice at 4 months of age. (n): number of events. Results are expressed as a mean ± Standard error of the mean (SEM). *p<0.05; **p<0.01; ****p<0.0001.

	**WT**	**GADD45A$^{-/-}$**
**Locomotor activity (cm)**	1476.18 ± 85.68	1870.07 ± 118.8 *
**Rearings (n)**	4.7 ± 1.08	31.17 ± 2.41 ****
**Time in Zone (%)- Border**	61.93 ± 5.42	79.63 ± 1.83 **
